# Identification, characterization and purification of porcine Quiescin Q6-Sulfydryl Oxidase 2 protein

**DOI:** 10.1186/s12917-017-1125-1

**Published:** 2017-06-29

**Authors:** Yu-Wen Kuo, Radhika Joshi, Tse-En Wang, Hui-Wen Chang, Sheng-Hsiang Li, Chun-Ni Hsiao, Pei-Shiue Jason Tsai

**Affiliations:** 1 Department of Veterinary Medicine, National Taiwan University, No. 1, Sec. 4, Roosevelt Rd, 10617 Taipei, Taiwan; 2 Graduate Institute of Veterinary Medicine, National Taiwan University, No. 1, Sec. 4, Roosevelt Rd, 10617 Taipei, Taiwan; 3 Graduate Institute of Molecular and Comparative Pathobiology, National Taiwan University, No. 1, Sec. 4, Roosevelt Rd, 10617 Taipei, Taiwan; 4 Department of Medical Research, Mackay Memorial Hospital, No. 92, Section 2, Zhongshan N. Rd, 251 Tamshui, Taipei, Taiwan; 5 Shui-Po International Certification Boar Semen Station, No. 71-115, 732 Tainan, Taiwan; 6 Research Center for Developmental Biology and Regenerative Medicine, National Taiwan University, No. 1, Sec. 4, Roosevelt Rd, 10617 Taipei, Taiwan

**Keywords:** Epididymis, Porcine, Quiescin Q6-Sulfydryl Oxidase, Seminal vesicle

## Abstract

**Background:**

Post-spermiogenesis membrane surface modifications rely on molecules present in the reproductive tracts. Two isoforms (isoform 1 and 2) from Quiescin Q6-Sulfydryl Oxidase protein family have been identified in the male reproductive tract of rodent species. However, unlike isoform 1, scarce information is available for isoform 2, likely due to its lower expression level and lack of proper purification methods to obtain sufficient protein quantity for further assays.

**Results:**

This study demonstrated the presence of short and long forms of Quiescin Q6-Sulfydryl Oxidase 2 in boar, likely representing the secretory (short form) and transmembrane (long form) forms of Quiescin Q6-Sulfydryl Oxidase 2. Immunohistochemistry studies revealed the presence of Quiescin Q6-Sulfydryl Oxidase 2 in a broad range of porcine tissues; the pronounced vesicle-contained Quiescin Q6-Sulfydryl Oxidase 2 at the apical region of epididymis and seminal vesicles epithelium suggested its involvement in sperm physiology and its participation in semen formation. The majority of porcine Quiescin Q6-Sulfydryl Oxidase 2 could be purified via either antibody affinity column or be salted out using 10%–40% ammonium sulfate. Higher amount of low molecular weight Quiescin Q6-Sulfydryl Oxidase 2 observed in the seminal vesicle likely represents the secretory form of Quiescin Q6-Sulfydryl Oxidase 2 and reflects an exuberant secretory activity in this organ.

**Conclusions:**

We demonstrated for the first time, the presence of Quiescin Q6-Sulfydryl Oxidase 2 in porcine species; moreover, two forms of Quiescin Q6-Sulfydryl Oxidase 2 were identified and exhibited distinct molecular weights and properties during protein purification processes. This study also provided feasible Quiescin Q6-Sulfydryl Oxidase 2 purification methods from slaughterhouse materials that could potentially allow obtaining sufficient amount of Quiescin Q6-Sulfydryl Oxidase 2 for future functional investigations.

## Background

The formation of a functional male gamete is an outcome of complex events involving biochemical, physiological and morphological modifications of the spermatocyte. Spermatogenesis starts in the testis with multiple mitotic divisions, followed by two subsequent cycles of meiotic division, resulting in the formation of a haploid spermatocyte [1]. Although the diploid spermatogonium has transited to a haploid spermatocyte, it is still considered immature due to the lack of forward movement and oocyte recognition abilities [2, 3]. Once the spermatozoa leave the seminiferous tubules, post-spermiogenetic maturation continues in the epididymis. Epididymal maturation involves several sequential interactions of the spermatozoa with the surrounding luminal microenvironment [4]. The exposure of spermatozoa to a dynamic milieu of the luminal fluid in different epididymal segments alters the net surface charge, membrane protein, phospholipid and fatty acid compositions as well as their immunoreactivity, and adenylate cyclase activity [5]. Many of these processes are thought to improve the structural integrity of the sperm membrane and to increase or to regulate the fertilization ability of the spermatozoa.

Upon ejaculation, spermatozoa are mixed with secretions from the accessory glands (prostate gland, vas deferens and seminal vesicle). Amongst constituent components that form seminal plasma, proteins are of most important ones that directly or indirectly regulate sperm functions and activities. One of the protein families that have been identified in the seminal plasma is the Quiescin Q6-Sulfydryl Oxidase (QSOX) family. QSOX protein was first discovered by Otrowoski and co-workers from rat seminal vesicles back in 1979 [6]. It was characterized as a flavoprotein responsible for converting sulfhydryl containing substances to corresponding disulfides at the expense of molecular oxygen and generating hydrogen peroxidase as shown in equation: 2R–SH + O_2_ **→** R-S = S-R + H_2_O_2_ [6].

The substrates of QSOXs range from small thiol compounds such as dithiothreitol (DTT) to thiol groups in proteins. The formation of the covalent linkages between thiol groups is considered as a reversible post-translational modification process commonly observed in secreted proteins. Intra molecular disulfide bonds are required for proper protein folding, function and its stability [7]. Moreover, they are also crucial for linking different proteins together through their sulfhydryl groups [7]. Two genes, QSOX1 and QSOX2 are encoded with the QSOX proteins. In the human genome, QSOX1 gene is located on chromosome 1. QSOX1 was first identified from the fibroblasts of human lung [8, 9] and Benayoun et al. later revealed its sequence by the use of adult rat seminal vesicle [10]. In different animal species, an alternative splicing further generates a long (QSOX1-L, also named QSOX1a) and a short (QSOX1-S, also known as QSOX1b) transcripts [11–14]. In contrast to QSOX1a which contains an additional transmembrane domain, QSOX1b appears as a shorter form due to the alternative splicing [13, 15, 16]. The second gene that encodes a 75 kDa QSOX2 protein is located on chromosome 9. QSOX1-L, QSOX1-S and QSOX2 constitute of two thioredoxin domains at the N-terminus followed by a helix-rich region (function yet to be identified) and a C-terminal Erv/ALR domain. The last domain represents the oxidative site for the catalysis of the thiol/disulfide redox pool to molecular oxygen via/through FAD cofactor [8, 17–20]. QSOX1 and QSOX2 share 40% identity in their primary structure and 68% in their functional ERV1 and thioredoxin domains [12]. QSOX1 has been detected both extra- and intracellularly (in mitochondria), and is related to protein folding, elaboration of extracellular matrix, redox regulation, and cell cycle control [11, 13, 21, 22]; whereas QSOX2 was observed on the plasma membrane and in the nucleus [12, 16], showing lower expression level than QSOX1 in most of the human tissues [22]. In male genital tract, high levels of the sulfhydryl oxidase activity were first described in the secretions from the epididymis and the seminal vesicle of rats and hamsters [23]. They speculated that the sulfhydryl oxidase might be involved in protecting spermatozoa from endogenous sulfhydryl groups and thereby preserving sperm structure and function [23–25].

Interestingly, despite an early identification (from human neuroblastoma cells) of QSOX2 protein and its associated gene [16], up to date, limited information is available on QSOX2 protein. This is likely due to (1) its low expression levels when compared with QSOX1 and (2) difficulties on purification to obtain sufficient amount of QSOX2 protein for further functional studies. In this study, we first examined the presence and tissue distribution of QSOX2 in boar, of which the species with a relatively large quantity (as compared with both rodent and human species) of materials can be obtained. We further explored the possibilities for QSOX2 purification from porcine seminal vesicle and epididymis due to their easy accessibility and relative large volume (both organs and their secreted fluid) available from slaughterhouse. In this study, we not only demonstrated for the first time, a pronounced and specific localization of QSOX2 on the epithelium of porcine reproductive tract, but also provided an alternative approach for large scale QSOX2 purification from porcine seminal vesicle and epididymis. Our study may provide new opportunities for obtaining sufficient amount of QSOX2 from discarded slaughterhouse materials for further functional characterizations/assays.

## Methods

### Chemicals, reagents, antibodies

Chemicals and reagents were obtained from Sigma-Aldrich (St. Louis, MO, USA) unless otherwise stated. Rabbit polyclonal anti-QSOX1 and anti-QSOX2antibodies were purchased from Abcam (Cambridge, UK). Magnetic Dynabeads®Protein A Immunoprecipitation Kit was obtained from Thermo Fisher Scientific (MA, USA).

### Animals

Tissues used in this study were obtained from slaughterhouse materials upon routine visits for health and meat production inspections under the guidance of Council of Agriculture, Taiwan. Tissue sampling for pigs was carried out under the supervision of a certified slaughterhouse veterinarian and under IACUC protocol (NTU-103-EL-86). For porcine analyses, materials from eight male Landrace pigs, aged between 2 to 2.5 years were collected in late March. Samples were collected immediately after slaughter. For murine analyses, 8-week-old male ICR mice were purchased from National Laboratory Animal Center, all experiments were approved by IACUC committee of National Taiwan University (NTU-103-EL-86, NTU104-EL-00081).

### Tissue preparation

For porcine analyses, reproductive (i.e. testis, seminal vesicles and the caput epididymis) and non-reproductive organs (i.e. heart, lung, liver, spleen, small intestine, kidney, and skeletal muscle) were collected from slaughterhouse. Tissue samples were immediately transferred onto dry ice before further storage at −80 °C. For murine analyses, seminal vesicles and caput epididymis from 8-week-old male ICR mice (*n* = 4) were collected. After collection, both porcine and murine samples were divided for immunohistochemistry, immunoblotting analysis, and protein purification.

### Immunohistochemistry

For histo-chemical analysis, representative tissue samples were collected, fixed in 10% neutral buffered formalin, processed routinely for paraffin blocks and sectioned at 10 μm. Hematoxylin and eosin (H&E) stain was used for general histological and morphological examinations. For IHC staining, tissue sections were deparaffinized with 100% xylene and rehydrated with 100%–80% ethanol. Antigen retrieval was carried out by heating tissue sections in 10 mM citrate buffer (pH 6.0) using regular microwave at 95 °C for 2 cycles. Endogenous peroxidase was subsequently removed by incubating tissue sections with 3% (*v*/v) hydrogen peroxide (H_2_O_2_) for 30 min at room temperature (RT). To minimize non-specific signals, sections were incubated with 2.5% filtered normal goat serum (NGS) diluted in Tris-buffered saline (TBS, 5 mM Tris, 250 mM sucrose, pH 7.4) for 30 min, RT. Anti-QSOX2 antibody was used at a dilution of 1:100 and anti-QSOX1 antibody was in 1:200 dilution as recommended by the company datasheet. Polymer-HRP reagent (BioGenex HRP kit) was used as secondary antibody after intensive washes of sections with TBS. Reactions were developed with 2% diaminobenzidine (DAB, Dako Real DAB + Chromogen) for 10 min and slides were counter stained with hematoxylin (Muto Pure Chemicals Co. LTD, Tokyo, JP) for 30 s.

### Immuno-blotting

Immunoblotting procedures were followed as previously described [26]. For protein homogenate preparation, materials were homogenized on ice in tissue homogenization buffer (250 mM sucrose, 1 mM EDTA, 20 mM Tris/Hepes, 1% Triton X-100, pH 7.4) using glass homogenizer in the presence of protease inhibitor cocktail tablet (EDTA free, Roche, Mannheim, Germany). Protein concentrations were measured according to the Pierce® BCA Protein Assay Kit (Pierce Biotechnology, Rockford, IL, USA) and were stored at −80 °C until use. Equivalent amount of total protein extract (μg) was re-suspended with an appropriate volume of Lithium dodecyl sulfate (LDS) loading buffer (Invitrogen) in the presence of reducing agent (50 mM DTT), samples were heated in a 95 °C dry bath for 10 min and cooled on ice before loading on gels. Bio-Rad Mini-PROTEIN® electrophoresis system was used (Bio-Rad Laboratories Ltd., Hertfordshire, DX) and standard manufactory protocol was followed. Proteins were separated by SDS-PAGE (gradient T-Pro EZ Gel Solution, T-Pro Biotechnology, NTC, TW) and wet-blotted onto a PVDF membrane (Immobilon-P, Millipore, Billerica, MA, USA). After blocking for 1 h with blocking buffer (5 mM Tris, 250 mM sucrose, pH 7.4 with 0.05% *v*/v Tween-20 [TBST], supplemented with 5% milk powder) at room temperature, blots were incubated with primary antibody (1:250 diluted in TBST) for overnight at 4 °C. After three times washing with TBST, secondary antibody was subsequently added and blots were further incubated at RT for 1 h. After rinsing with TBST, protein was visualized by using chemiluminescence (Merck, Ltd., TW). When necessary, blots were stripped (Thermo Scientific Restore Western Blot Stripping Buffer) and re-probed for other proteins of interests.

### Ammonium sulfate protein precipitation and Immunoprecipitation (IP)

Ammonium sulfate ((NH_4_)_2_SO_4_) was used for total protein precipitation from tissue homogenates of epididymis and seminal vesicle mixture, procedures were followed by company’s instructions (BioVision Inc.). Cells or tissue debris were span down by centrifugation at 3000 g for 10 min prior to precipitation processes. Supernatant was mixed in 1:1 ratio with phosphate buffered saline (PBS) in the presence of 1 M Tris-HCl. Proteins were salted out using 10%, 25%, 40%, 60%, 80% and saturated (100%) ammonium sulfate in this study. Sample homogenates were mixed and stirred with different percentages of ammonium sulfate for 15 min and centrifuged at 15,000 g (20 min at 4 °C). Protein pellets obtained from each ammonium sulfate concentration were re-dissolved in PBS and further quantified for total protein concentration as described earlier.

Immunoprecipitation was carried out according to company’s instructions. In brief, 1.8 μg of anti-QSOX2 antibody was first diluted in 200 μl of filtered antibody binding buffer (provided in the kit), mixed thoroughly with 50 μl Magnetic Protein A Dynabeads® on an end-to-end rotor for 30 min at RT to form beads-Ab complex. After washing with washing buffer (provided in the kit), tissue homogenate mixtures (containing 1 mg of total protein in 500 μl volume) were incubated with beads-Ab complex for 1 h at RT for allowing QSOX2 to bind to the preformed beads-Ab complex. Beads-Ab-QSOX2 complexes were subsequently separated from unbound proteins and supernatant on the magnet holder. Elution of the target antigen (QSOX2) was followed by adding 30 μl of elution buffer (provided in the kit) for 10 min at RT. Elute was quantified for protein concentration and stored at −80 °C in the presence of protease inhibitor as described above.

## Results

### Immunohistochemistry analyses demonstrated the presence of QSOX2 in various porcine tissues

**Fig. 1:**
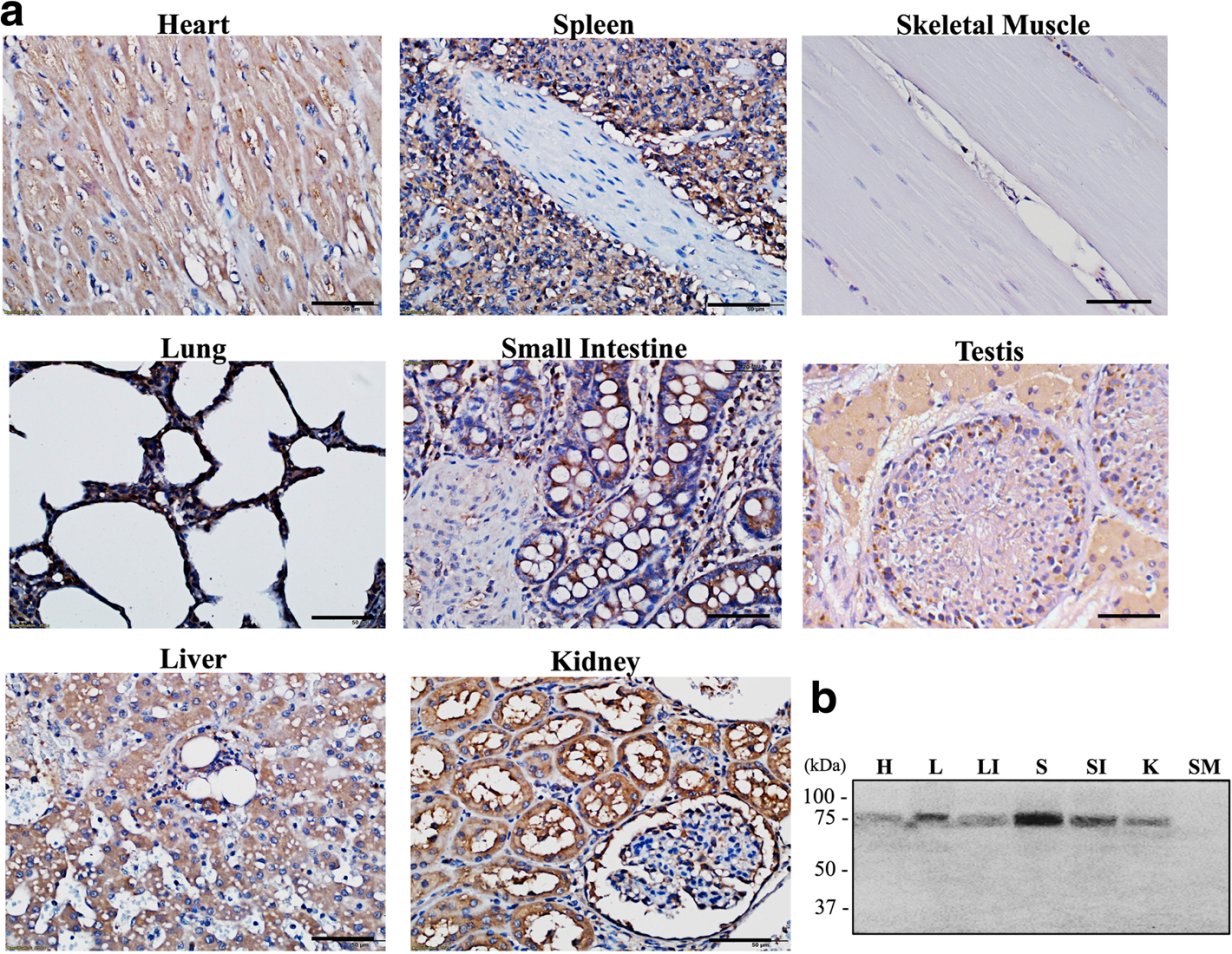
Tissue distribution of porcine QSOX2 IHC and Western blot analysis of various boar tissues using rabbit polyclonal anti-QSOX2 antibody show. **a** Most of the tissues examined showed a homogenous distribution. **b** A single band was detected at 75kD in most of the tissue homogenates. Heart (*H*) showed the weakest signal, moderate intensities were detected in lung (*L*), liver (*LI*), small intestine (*SI*) and in kidney (*K*) when 30 μg of total protein was loaded on gel. A relative strong signal could be detected in spleen (*S*) with skeletal muscle (*SM*) appeared negative for both immunohistochemistry and western-blot detection in boars. Bar = 50 μm

To investigate the presence and tissue distribution of QSOX2 in porcine, IHC studies were carried out. Our results demonstrated that QSOX2 was present in most of the tissues tested including in heart, lung, liver, spleen, small intestine and kidney. In line with previous reports from rodents or human, no signal can be detected in skeletal muscle; however, unlike in humans, we observed a positive signal in porcine heart (Fig. 1a), even though its expression level is low. To further verify signals detected by immunohistochemistry studies, Western blotting analyses were carried out as described in the methods and material section. In agreement with results from immunohistochemical studies, a distinct band at 75kD was detected in most of the tissues examined besides skeletal muscle (SM). Weak signals were detected in heart (H) and liver (LI), whereas lung (L), spleen (S), small intestine (SI) and kidney (K) showed significantly strong signals (Fig. 1b). This is, to our knowledge, the first report on the presence of QSOX2 in porcine species (*Sus scrofa*).

### QSOX2 appeared specifically in a vacuole-like structure at the apical region of the epithelium in the reproductive tract

**Fig. 2:**
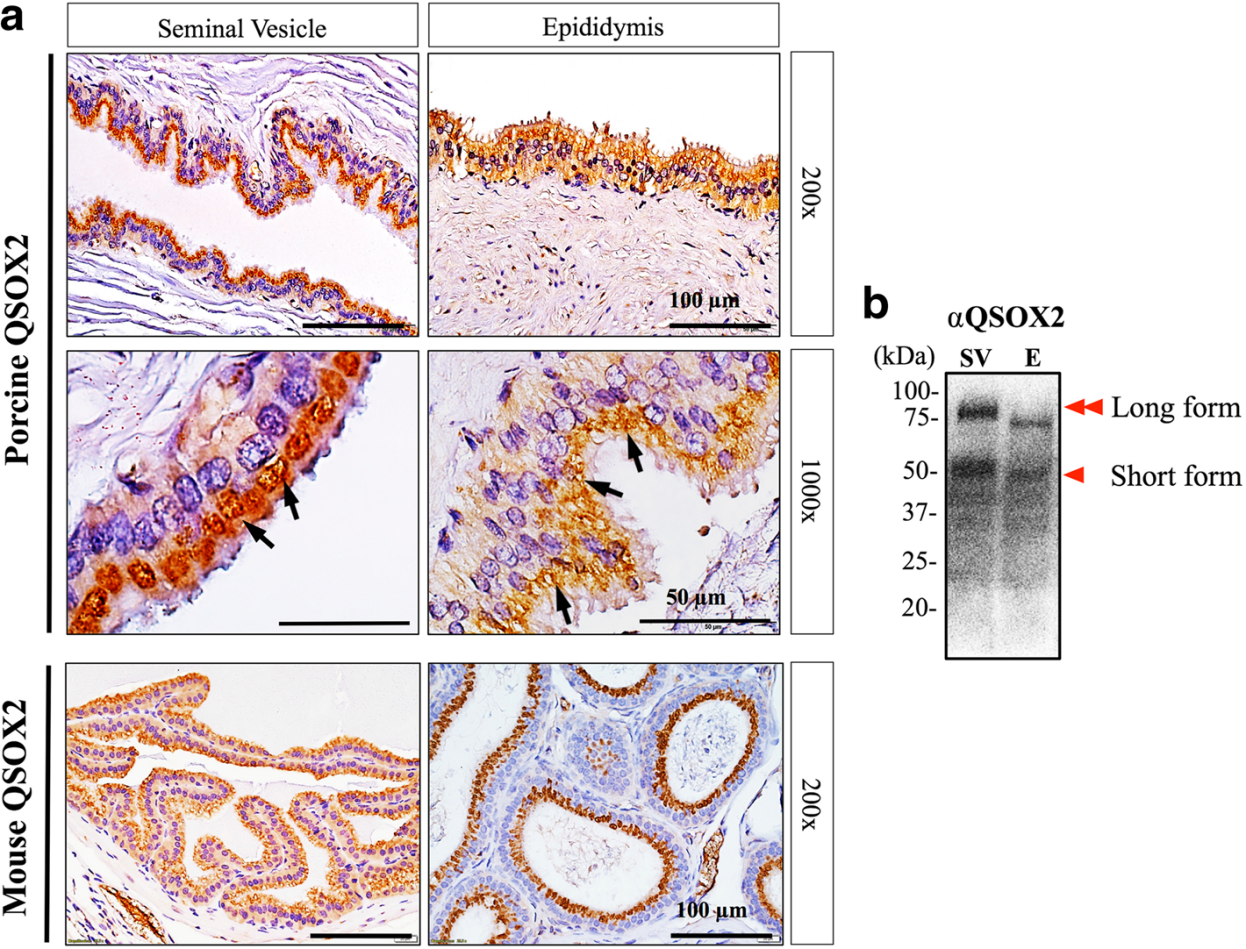
Immunohistochemical characterization of QSOX2 in boar seminal vesicle and epididymis. **a** Representative immunohistochemistry images demonstrated a pronounced epithelium signal in boar reproductive organs. The prominent vesicle-like structure (marked with *arrows*) contained QSOX2 was observed specifically at the apical ridge of epithelial cells in porcine reproductive tract. This staining pattern was similar to those in mouse. **b** Western-Blotting analysis showed that two distinct bands at 50 kDa (marked with one arrowhead) and 75 kDa (marked with two *arrowheads*) can be detected in both boar seminal vesicle (SV) and epididymis (E) suggesting the presence of both long (75 kDa) and short (50 kDa) forms of QSOX2

**Fig. 3:**
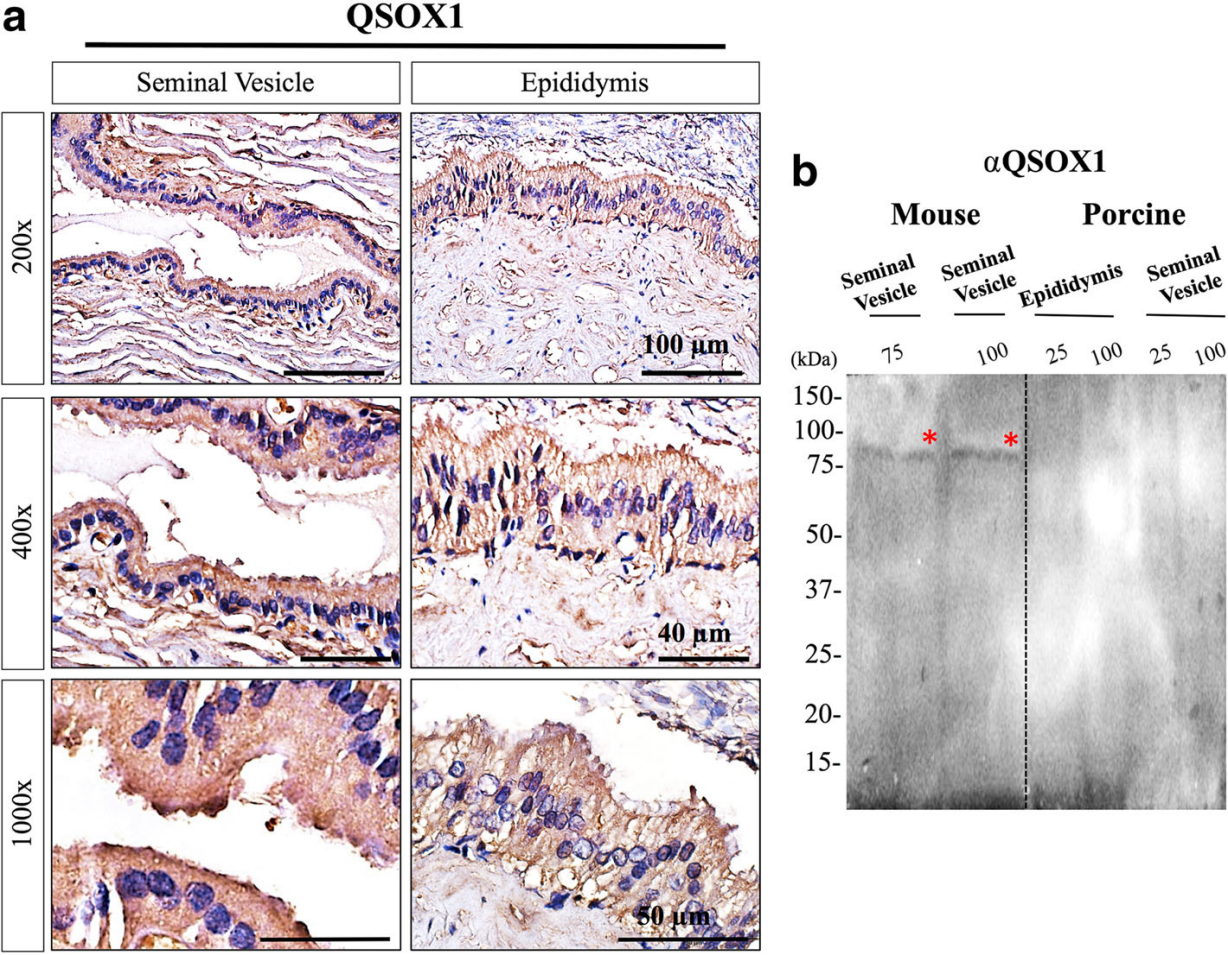
Immunohistochemical characterizations of QSOX1 in mouse and boar. **a** Representative immunohistochemistry images showed that unlike specific epithelium staining of QSOX2, a weak and homogenous staining pattern was detected in both epithelium and interstitial area of porcine seminal vesicle and epididymis. **b** Western-Blotting analysis showed positive signal at ~82 kDa in mouse seminal vesicles and epididymis; however, no signal can be detected in any of the porcine tissue examined

As early discovery of QSOX2 was made from male reproductive tracts of rodent species [27], we focused on examining the presence of QSOX2 in porcine reproductive organs, namely seminal vesicle and epididymis. Unlike homogenous distribution observed in other non-reproductive organs (Fig. 1a), QSOX2 appeared in a vacuole-like structure located at the apical side of the reproductive tract epithelium (Fig. 2a. *marked with arrows*). This pronounced and specific epithelium staining pattern is similar to our observations in mouse reproductive tracts (Fig. 2a, *lower panels*). In contrast to a single band detected in other tissue homogenates from non-reproductive organs, two distinct bands at 50kD and 75kD were detected in both epididymis and seminal vesicles (Fig. 2b). This likely represents the long (marked with two arrow heads) and the short (marked with one arrow head) form of porcine QSOX2. To our knowledge, both QSOX1 and QSOX2 have been reported in other mammalian species; however, up to date, no information has been reported in boar. In most of the mammalian species, QSOX1 and QSOX2 share 38% (in human) - 47% (in mouse) similarity in amino acid sequence. We next compared the differences between porcine QSOX1 and QSOX2 by performing immunohistochemistry and Western-Blotting analyses. Unlike epithelium-specific staining pattern observed for porcine QSOX2, porcine QSOX1 was expressed weakly and homogenously in both epithelium and interstitial area of porcine seminal vesicle and epididymis (Fig. 3a), without noticeable vacuole-like structures as observed for QSOX2. From Western-Blot analysis of mouse seminal vesicle, a positive signal was observed at ~82 kDa as predicted by mouse QSOX1 sequence; however, no signal can be detected in the porcine tissue homogenates of seminal vesicle and epididymis (Fig. 3b).

### Porcine QSOX2 purification via ammonium sulfate and immunoprecipitation (IP)

**Fig. 4:**
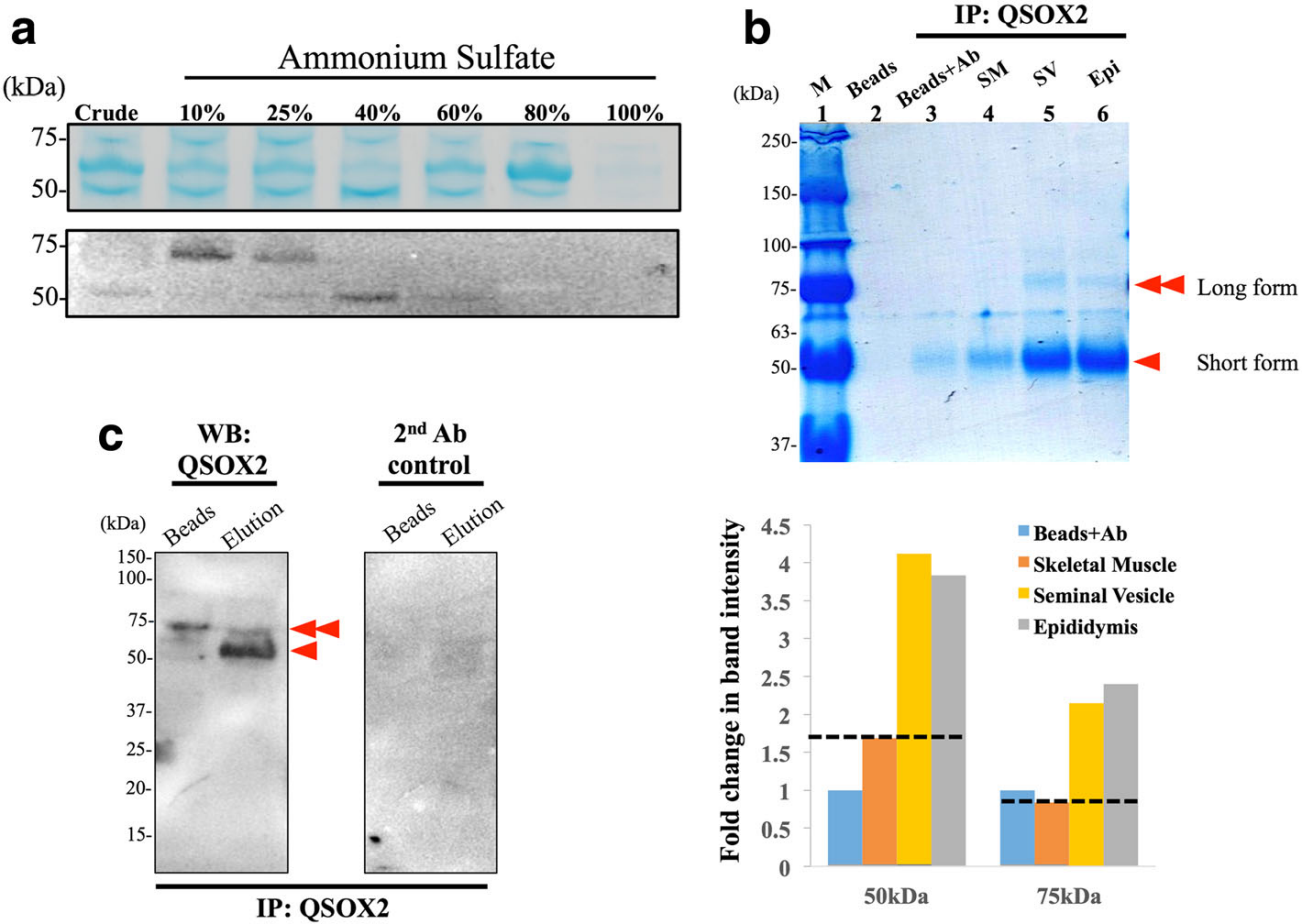
Two forms of porcine QSOX2 can be purified via immunoprecipitation (IP). **a** A total protein precipitation carried out by different percentages (10–100%) of ammonium sulfate indicated that boar QSOX2 is mostly precipitated with 10%–40% ammonium sulfate. **b** SDS page stained with Coomassie blue indicated that anti-QSOX2 antibody could recognize and precipitate both long (*marked with 2 arrowheads*) and short (*marked with 1 arrowheads*) form of porcine QSOX2. After subtracting band intensity detected in control lanes (skeletal muscle, lane 4), we detected a 2.2 to 2.4-fold increase in band intensity at 50 kDa and a 1.3 to 1.6-fold increase at 75 kDa. **c** Western-Blotting analysis further confirmed the presence of both 50kD and 75kD QSOX2 in the eluted fraction. However, the majority of the long form QSOX2 remained on beads

From data presented above, we considered QSOX2 from male reproductive tracts might share high level of similarities in both tissue distribution (epithelium-specific) and biochemical properties (contained both long and short form), which are not the same as the rest of tissues examined (Fig. 1). To increase the yield of QSOX2 protein purification, we therefore combined tissue homogenates of seminal vesicles and epididymis as our starting material for ammonium sulfate precipitation. We first performed a total protein precipitation using ammonium sulfate ((NH_4_)_2_SO_4_). From Fig. 4a (*upper panel*), proteins were mostly precipitated when 10%–80% ammonium sulfate were used. Western-Blotting analysis of the precipitates revealed that the long form QSOX2 (at 75 kDa) could be precipitated using 10%–25% (NH_4_)_2_SO_4_ while the short form QSOX2 (at 50 kDa) could be precipitated using 40% (NH_4_)_2_SO_4_. We further performed immunoprecipitation using commercially available anti-QSOX2 antibody. From SDS page of immunoprecipitated samples, clear bands were visible at 75 kDa, 68 kDa and 50 kDa (Fig. 4b). In our Western blot, when compared to our experimental controls (i.e. beads only [lane 2], beads with anti-QSOX2 antibody without porcine samples [lane 3]), band intensities at both 50 kDa (marked with one arrow head) and 75 kDa (marked with two arrow heads) were much stronger with seminal vesicle (lane 5) or epididymis (lane 6) samples/elutes (Fig. 4b, *upper panel*). As skeletal muscle was considered negative for QSOX2 in porcine species based on our data presented above (Fig. 1a, b), we calculated a relative band intensity using skeletal muscle (orange bars) for background subtraction. As shown in Fig. 4b (*lower panel*), we detected a 2.4 and 2.2-fold increase in 50 kDa signal in seminal vesicle (yellow bar) and epididymis (grey bar), respectively as compared to the signal detected in skeletal muscle. For long form porcine QSOX2 detected at 75 kDa, a 1.3 and 1.6 fold increase was measured in seminal vesicle (yellow bar) and epididymis (grey bar), respectively. This was further confirmed by Western-Blotting analysis from the seminal vesicle and epididymis tissue mixture immuno-precipitates, which showed two major bands at 50 kDa and 75 kDa in the eluted fraction. Interestingly, under the same elution condition, a large portion of long form QSOX2 remained on the bead while most of the short form QSOX2 was eluted suggesting biochemical differences between two porcine QSOX2 isoforms (Fig. 4c).

## Discussion

After the spermatozoa are liberated from testis, membrane surface modifications continue with constant re- and de-coating events induced by male accessory fluid [28, 29]. The exposure of spermatozoa to the luminal fluid of epididymis during sperm transition not only alters the compositions of sperm membrane surface protein and lipids [5, 30], but also regulates sperm activities and functions through fine adjustment of epididymal luminal pH [31–34]. After ejaculation, spermatozoa are mixed with seminal plasma, the seminal plasma contains proteins, lipids, amino acids, carbohydrates like fructose and other elemental substances that not only provide essential nutrients and metabolites for sperm motility, but also facilitate the regulation of sperm functions with “de-capacitation” factors [35–37]; thereby preventing pre-mature capacitation and acrosome reaction prior to their encounter with the oocyte in the oviduct.

In this study, we demonstrated for the first time, the presence of Quiescin Q6-Sulfydryl Oxidase 2 (QSOX2) in porcine species and further showed its ubiquitous tissue distributions in heart, lung, liver, spleen, small intestine, kidney, testis, seminal vesicle and epididymis, except in skeletal muscle (Fig. 1). This wide-ranged tissue distribution is in accordance with previous reports of QSOXs in human and rodent species [13, 15, 27, 38]. Of particular interest is, the pronounced epithelial expression of QSOX2 in male reproductive organs (Fig. 2). Unlike QSOX2 observed in non-reproductive tissues, QSOX2 present in male reproductive tract were detected in a vesicle-like structure at the apical side of the epithelial cells. This specific cellular arrangement suggests that QSOX2 might later be secreted into the lumen and may participate in the formation of seminal fluid and epididymal fluid via specific but, yet to be identified regulatory mechanism. This speculation is supported by our Western-Blotting analysis in which we detected two forms of QSOX2 from the seminal vesicle and epididymal tissue homogenates (Fig. 2b). Our observation is in line with the current knowledge of QSOX1 protein having both long (isoform a, with transmembrane domain) and a short (isoform b, without transmembrane domain) [11–14]. Based on mRNA sequences, two isoforms of QSOX2 protein have earlier been predicted in some species like mouse, rat, *Cebuscapucinus imitator*, white headed capuchin, chimpanzee and gray short-tailed opossum (NCBI data base). It is reasonable to predict that in boars, tissues or organs with less or no secretion function may consist mainly of transmembrane domain containing QSOX2, exhibiting a higher protein molecular weight at ~75 kDa; whereas organs with exuberant secretory activities like seminal vesicles and epididymis contain besides transmembrane QSOX2, an additional secretory form of QSOX2 with a smaller molecular weight due to the lack of transmembrane domain as seen from our Western-blotting analysis (Figs. 2 and 4). Therefore, future studies focusing on the underlying regulatory mechanisms of QSOX2 secretion may help to reveal the function of QSOX2 and its involvement in reproductive physiology.

Functional analyses of QSOX2 rely on the purity of the protein. However, compared with QSOX1 expression, QSOX2 expresses a much lower level in most of the human tissues [22]. To overcome this natural limitation, we explored the possibilities of purifying porcine QSOX2 from male reproductive organs (i.e. seminal vesicle and epididymis). In most parts of the world, boar seminal vesicles and epididymis are treated as slaughterhouse wastes, thereby allowing easy access to sufficient starting material for protein isolation. Our data supports the feasibility of QSOX2 purification by either ammonium sulfate or anti-QSOX2 antibody, using the slaughterhouse wastes as starting materials. We saw QSOX2 could mostly be precipitated with 10%–40% ammonium sulfate concentration. Intriguingly, we observed differences in protein precipitation efficiency between long and short form of porcine QSOX2 by the use of ammonium sulfate. These reflect the natural biochemical differences between two forms of QSOX2. Ammonium sulfate is known to precipitate proteins due to its high water solubility. Proteins with lower water solubility (e.g. transmembrane proteins, like long form QSOX2) will be precipitated easier/earlier than those of high water soluble proteins (e.g. secretory proteins, like short form QSOX2), thereby proving our observation of high molecular weight QSOX2, but not low molecular weight QSOX2 been detected under 10% ammonium sulfate.

As protein composition of porcine seminal fluid is complex and contains many proteins other than QSOX2 [39, 40], to reach a higher purity of QSOX2, we further performed immunoprecipitation using commercially available anti-QSOX2 antibody. As shown in Fig. 4b and c, with immunoprecipitation, we enhanced QSOX2 signals 1.3–2.4-fold more than background signals. Interestingly, we observed with this purification approach, a higher amount of low molecular weight QSOX2 (likely the secretory form of QSOX2). Based on information available from antibody producing company, the immunogen of this antibody resides at the internal sequence amino acids 504–633 of human QSOX2 which is the region before transmembrane domain; therefore, it is likely that by the use of this antibody, we pulled down both short and long forms of porcine QSOX2. Since seminal vesicle and epididymis are organs with active secretory activities, a relatively higher proportion of short form QSOX2 is expected to be present in these organs.

In conclusion, in this study, we showed for the first time, the presence of QSOX2 in porcine species, and porcine QSOX2 exhibits two isoforms which may represent transmembrane (long form, high molecular weight) and secretory (short form, low molecular weight) forms of QSOX2; moreover, both QSOX2 can further be purified via immunoprecipitation using specific antibody. Our data not only provides evidence on the identification of QSOX2 in porcine species, but also indicates a new source and approach for QSOX2 purification for future functional analyses.

## Conclusions

QSOX2 is enriched in porcine epididymis and seminal vesicles and obtaining sufficient amount of QSOX2 is critical for in-depth functional analyses of QSOX2. Porcine materials from the slaughterhouse serve as ideal sources for protein purification without additional sacrifice of individuals. Large-scale QSOX2 protein purification and accompanied functional studies will shed lights on functional relevance of QSOX2 on sperm physiology.

## Abbreviations

EDTA: Ethylenediaminetetraacetic acid
Erv/ALR: Augmentor of Liver Regeneration
H: Heart
HRP: Horse Raddish Peroxidase
IACUC: Institutional Animal Care and Use Committee
IP: Immunoprecipitation
K: Kidney
L: Lungs
LDS: Lithium Dodecyl Sulfate
Li: Liver
NGS: Normal Goat Serum
PBS: Phosphate Buffered Saline
PVDF: Polyvinilydene Fluoride
QSOX: Quescin Q-6 Sulfydryl Oxidase
QSOX-a: Quescin Q-6 Sulfydryl Oxidase-a
QSOX-b: Quescin Q-6 Sulfydryl Oxidase-b
QSOX-L: Quescin Q-6 Sulfydryl Oxidase- Long form
QSOX-S: Quescin Q-6 Sulfydryl Oxidase- Short form
S: Spleen
SI: Small Intestine
SM: Smooth muscle
T: Testis
TBS: Tris Buffered Saline
